# Determining Compressed Concrete Element Limit States Based on the Widths and Depths of Cracks Caused by Transverse Deformations

**DOI:** 10.3390/ma17020355

**Published:** 2024-01-10

**Authors:** Iakov Iskhakov, Ilya Frolov, Yuri Ribakov

**Affiliations:** Department of Civil Engineering, Ariel University, Ariel 40700, Israel

**Keywords:** experimental investigations, numerical investigations, transverse deformations, limit states, crack width, crack depth, compressed concrete, stress–strain diagram

## Abstract

In the modern theory of compressed concrete elements, the most attention is paid to longitudinal deformations, whereas transverse ones are rarely considered and just within Poisson’s coefficient limits (i.e., elastic concrete behavior in the transverse direction). However, transverse deformations significantly develop beyond the limits corresponding to Poisson’s coefficient, where they lead to longitudinal crack initiation and development. In-depth experimental and numerical investigations of transverse deformations in the inelastic stage showed that it is necessary to consider crack propagation. The present study proposes simultaneous consideration of longitudinal and transverse deformations, as well as the appearance of cracks and their widths and depths. This allowed us to obtain a complete compressed concrete element behavior pattern at all performance stages in two types of limit states (based on longitudinal and transverse deformations). Consequently, new ultimate limit states by the depth and width of cracks caused by transverse deformations are proposed to be included in modern design practices and codes.

## 1. Introduction

A history of compressed concrete investigation shows that, in the first research steps [1,2], in the 1970s and 1980s [3,4,5,6], and up to the present time [7,8,9], the main focus has been on deformations developing parallel to the load direction. Studies of uniaxially compressed concrete specimen behavior in the transverse direction are usually limited to the elastic performance stage. In this stage, transverse deformations are generally determined by Poisson’s coefficient [10,11,12,13]. Although many experimental investigations have been carried out to study concrete specimen behavior in the longitudinal direction, few of them have focused on transverse deformations out of the elastic limits. For example, in one study, a criterion for splitting crack formation and a constitutive model was presented to predict lateral deformation in confined concrete [14]. However, confined concrete is beyond the scope of this paper.

The authors have previously demonstrated the importance of using transverse deformations in addition to longitudinal ones for analyzing compressed concrete specimen behavior both in elastic and inelastic stages [15]. Seven 15 × 30 cm cylindrical specimens were tested under a constant loading rate of 0.2 MPa/s. The obtained results demonstrated the efficiency of using transverse deformations to reveal inelastic concrete behavior when it is not evident from longitudinal deformation analysis only.

Transverse deformations also more accurately indicate steel-fiber-reinforced high-strength concrete inelastic performance. Experimental results obtained previously by testing fourteen cylindrical specimens with 15 cm diameters and 30 cm heights subjected to static uniaxial loading confirmed the importance of using transverse deformations for the analysis of compressed concrete behavior.

Sixty cylindrical specimens with 10 cm diameters and 20 cm heights made of self-compacting normal-strength concrete with fine and coarse recycled concrete aggregate were subjected to monotonic loading, as well as to loading–unloading tests with increasing load magnitudes [16]. The obtained experimental data demonstrate the significantly more pronounced inelastic behavior of specimens in the transverse direction, which is evident from the stress–transverse deformation diagrams.

Using circumferential strains for the deep analysis of a rebar pull-out test on concrete specimens enabled the enhanced understanding of bonding resistance mechanisms [17]. For this purpose, twelve cylindrical specimens with diameters of 100, 150, and 200 mm and heights of 200 mm were tested. The obtained experimental results allowed for the development of a new theoretical three-dimensional model of specimen behavior during pull-out tests. It should be noted that, in this investigation, transverse cracks caused by longitudinal tensile deformations were studied, while in compressed concrete elements, longitudinal crack opening is caused by transverse deformations.

An experimental study on transverse deformations in prestressed reinforced concrete (RC) two-layer beams (TLBs) with spans of 8 m was conducted in [18]. One of the aims of this study was to improve concrete theory related to the Poisson ratio in the elastic and inelastic stages. This research was focused on concrete behavior in the compressed zone of bending RC elements. As a result, a new limit state for such elements was obtained. It allows for a more accurate prediction of crack development. This research was preceded by a long-term experimental program that included the investigation of 0.7 m long single-supported; 3 m long single-supported; continuous 4 m long two-span; and pre-stressed 3 m long TLBs. It should be noted that, in all these studies, transverse deformations were considered valuable indicators of effective concrete performance.

The available literature on the initiation and development of cracks in compressed concrete elements is rather limited. Mostly, crack width is considered the main parameter of limit state determination in bending RC elements [19]. Research on the relationship between crack width and depth has been conducted to investigate the importance of crack width implementation in RC element design; however, only the tensile zone of RC bending beams has been analyzed [20]. A study aimed at obtaining a constitutive theoretical model of compressed concrete behavior was conducted based on the existing experimental data, investigating the multiaxial stress states of cylindrical specimens under uniaxial, biaxial, and triaxial loading [21]. Different from the approach presented in this paper, transverse deformations were presented only as a part of the general damage process, which was modeled as a function of volumetric expansion and a degree of restraint provided by boundary conditions.

As one of the attempts to fill this gap, a theoretical model of longitudinal crack development in compressed concrete cylindrical specimens was proposed in [15]. This model allows to predict crack development during the entire loading process and includes the following three main stages:

– The stage before crack initiation corresponding to linear specimen behavior, $\varepsilon_{c\;trans}\leq \varepsilon_{c\;trans\;ul}$, where $\varepsilon_{c\;trans}$ is the transverse tensile deformation value, and $\varepsilon_{c\;trans\;ul}$ is the ultimate elastic transverse tensile deformation;
– The stage between $\varepsilon_{c\;trans\;ul}$—which determines the initiation of cracks—and the twofold ultimate elastic transverse tensile deformation value, $2\varepsilon_{c\;trans\;ul}$ (this stage corresponds to nonlinear specimen behavior);
– The stage of the specimen’s ultimate limit state (ULS), when transverse tensile deformations exceed $2\varepsilon_{c\;trans\;ul}$ (this stage corresponds to crack width development).

The above-described stages were described in previous experimental research [15]. Obviously, in the first stage, the cylindrical specimen section has an original undamaged circular shape. In the second stage, longitudinal cracks appear, and their depth increases under the external load. As a result, experiments show that an internal (undamaged) cylindrical core is formed.

The core section area before failure is equal to a part of the initial total cross-sectional one and can be calculated using the structural phenomenon [22], which determines the compressed concrete element limit state in the longitudinal direction. It was theoretically predicted and experimentally confirmed in [15] that, after reaching the ULS, the area of the specimen’s inner core is approximately half of the initial value. This experimental data will be used in the present study for a numerical step-by-step analysis of crack width and depth development in compressed concrete elements.

Although this study was performed for static loading, its outcomes can be used to interpret the reasons for a residential building collapse in Mexico during a strong earthquake in 2017 [23]. Transverse deformations that appeared in the entrance story columns at the beginning of the earthquake (the first 8–10 s) and developed during the earthquake (36 s) seemed to be the main reason for the collapse. As a result, longitudinal cracks developed in columns at the slab–column joint.

## 2. Research Aims, Scope, and Novelty

The main aim of the present study is to carry out a numerical step-by-step analysis of crack width and depth development due to transverse deformations in compressed concrete specimens under uniaxial loading. This study is based on the existing theoretical and experimental data on transverse deformation development [15]. Using this data and effective numerical methods allows for the complex analysis of compressed concrete behavior, which includes both longitudinal and transverse deformations, as well as longitudinal crack width and depth. This analysis resulted in a more accurate determination of crack width and depth at various loading stages. It has enabled the identification of a new parameter for compressed concrete element limit state definition. Additionally, a graph representing specimen behavior simultaneously in both directions is proposed. This novel approach became possible after proposing the “Concrete constitution”, which is theoretically based on the structural phenomenon and has been experimentally confirmed [22]. The proposed combination of theoretical and numerical modeling with experimental validation allows for a better understanding of concrete performance under various loading conditions. The research outcomes will enable us to improve the theory of compressed concrete elements in terms of limit states by transverse deformations and form a basis for the further development of existing design methods, as well as modern codes.

## 3. Background for Transverse Deformation Investigations

### 3.1. Longitudinal Deformations as a Background for Transverse Ones

**Figure 1:**
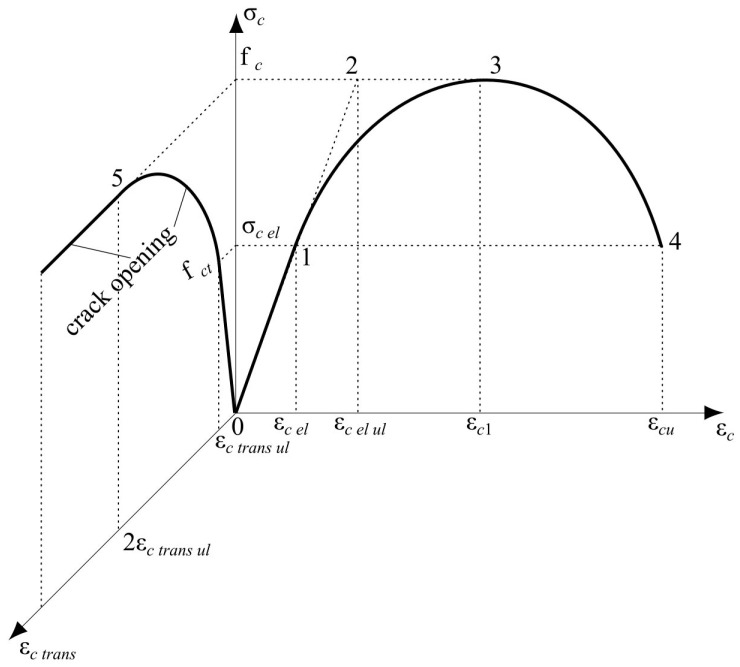
Compressed concrete stress–strain diagram: 1—the limit of the concrete section’s elastic stage; 2—the limit elastic potential of a section; 3—concrete strength; 4—the final point of the descending branch; 5—the border between nonlinear transverse deformation and compressed concrete ULS in the longitudinal direction.

A theoretical model for compressed concrete stress–strain relationships (including descending branch) without empirical coefficients is proposed below (Figure 1, $\sigma_{c}-\varepsilon_{c}$ axis). This model represents the above-mentioned “Concrete constitution”. The structural phenomenon [22] is one of the basic principles of this model and allows us to find the following features of the stress–strain diagram for concrete classes of up to C60 [24]:

– Maximum value of elastic deformations, $\varepsilon_{c\;el}=0.5‰$ (see point 1 in Figure 1);
– Deformation, $\varepsilon_{c\;el\;ul}=1‰$, corresponding to the limit elastic potential of concrete (see point 2 in Figure 1);
– Ultimate elastic stress, $\sigma_{c\;el}=0.5f_{c}$ (see point 1 in Figure 1);
– Diagram’s descending branch ends at $\sigma_{c\;ul}=0.5f_{c}$, which means that the descending branch is symmetric to the ascending one (see point 4 in Figure 1).

Other parameters determining compressed concrete behavior in the longitudinal direction are defined as in modern codes [24,25]:

$\varepsilon_{c1}$ is the compressive strain in concrete at peak stress, equal to 2‰ (see point 3 in Figure 1);
$\varepsilon_{cu}$ is the ultimate compressive strain in concrete, equal to 3.5‰.

Compressed concrete specimen behavior in the transverse direction is also presented in Figure 1 (see $\sigma_{c}-\varepsilon_{c\;trans}$ axis). According to the diagram, the following parameters are used:

$f_{ct}$ is concrete tensile strength in the transverse direction under longitudinal compression, corresponding to the appearance of longitudinal cracks in the specimen;
$\varepsilon_{c\;trans\;ul}$ is the ultimate elastic transverse strain determined by Poisson’s coefficient and is equal to 0.1‰ [15], which is approximately 20% of the corresponding longitudinal deformation value.

A detailed description of the main stages of transverse deformation development is provided in Section 4, below.

An experimental investigation of six cylindrical specimens subjected to axial loading was carried out to obtain a complete stress–strain relationship for normal-strength concrete [22]. The main aim of the investigation was to verify the theoretical stress–strain model, which forms the basis of the proper analysis of transverse deformations. The specimens were divided into two series and marked as Sp3.1, Sp3.2, Sp3.3, Sp4.1, Sp4.2, and Sp4.3. The concrete strength corresponded to classes C35/45 (ready-mix) and C20/25 (laboratory-produced) for each series. The obtained data showed good convergence with the theoretical data; therefore, it was used for further numerical investigation.

### 3.2. Numerical Investigation of the Stress–Strain Model Based on Experimental Results

**Figure 2:**
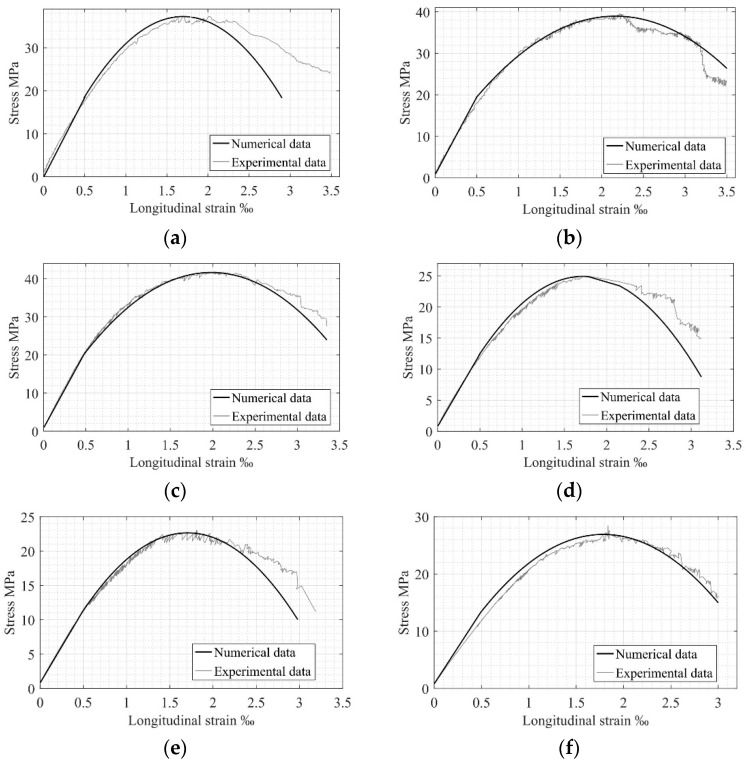
Comparison of experimental and numerical $\sigma_{c}-\varepsilon_{c}$ relationships for compressed concrete for specimens Sp3.1 (**a**); Sp3.2 (**b**); Sp3.3 (**c**); Sp4.1 (**d**); Sp4.2 (**e**); Sp4.3 (**f**).

The available experimental values of stresses and strains, as well as their dependencies [22], are used for numerical analysis, which is based on the known theoretical dependence (see Figure 1). A comparison of the corresponding experimental and numerical data using $\sigma_{c}-\varepsilon_{c}$ diagrams is presented in Figure 2. The results of comparing the numerical and experimental potential values of the stress–strain diagrams are presented in Table 1.

As is evident from Figure 2, there is a good agreement between the experimental and numerical $\sigma_{c}-\varepsilon_{c}$ relationships. Values of relative error, D, presented in Table 1, also show proper convergence between experimental and numerical data for the elastic potentials, describing the elastic stage of concrete behavior. Considering the inelastic stage, the maximum value of the relative error reaches 17%, which is satisfactory for materials like concrete. A comparison of the relationship between $U_{el}^{Num}$ and $U_{pl}^{Num}$, obtained from the experimental results and equal to 0.51, also confirms the structural phenomenon concept.

### 3.3. Complex Comparison of Theoretical, Experimental, and Numerical Results

**Figure 3:**
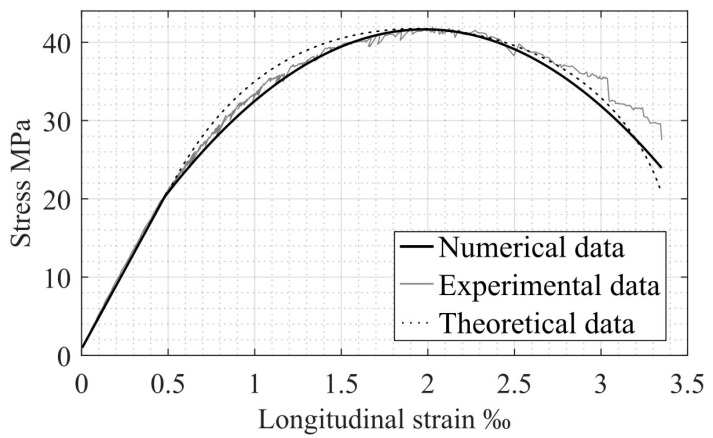
Comparison of numerical, theoretical, and experimental $\sigma_{c}-\varepsilon_{c}$ relationships for compressed concrete.

Stress–strain diagrams, based on the obtained numerical data, as well as on previously presented theoretical (see Figure 1) and experimental [22] stress–strain models, are combined in Figure 3. This approach enables a complex comparison of the results and leads to a more accurate identification of compressed concrete specimen performance in the longitudinal direction.

The obtained results show high convergence in all three graphs. Therefore, based on these data, further consideration of transverse deformations as a function of longitudinal ones is proposed.

## 4. Step-by-Step Analysis of Concrete Specimen Longitudinal Crack Width and Depth Based on Transverse Deformations

### 4.1. Background

As mentioned above, Figure 1 presents the performance of compressed concrete in both the longitudinal and transverse directions. It shows the relationship between the following three dependencies: longitudinal and transverse strains, $\varepsilon_{c\;long}$ and $\varepsilon_{c\;trans}$, and compression stresses, $\sigma_{c}$. Therefore, it is a 3D diagram, where only two main projections are presented for better clarity. According to Figure 1, the nonlinear concrete behavior in the transverse direction is presented by the ascending branch of the diagram, which starts at $f_{ct}$ and continues up to point 5, corresponding to the $2\varepsilon_{c\;trans\;ul}$ deformation value (see Figure 1, $\sigma_{c}-\varepsilon_{c\;trans}$ axis). This stage determines the longitudinal crack-opening process. After reaching concrete compressive strength, $f_{c}$ (see point 3 in the $\sigma_{c}-\varepsilon_{c}$ axis and point 5 in the $\sigma_{c}-\varepsilon_{c\;trans}$ axis), further crack development corresponds to the specimen failure process (the descending branch in longitudinal stresses, $\varepsilon_{c\;el}=2.0-3.5‰$).

**Figure 4:**
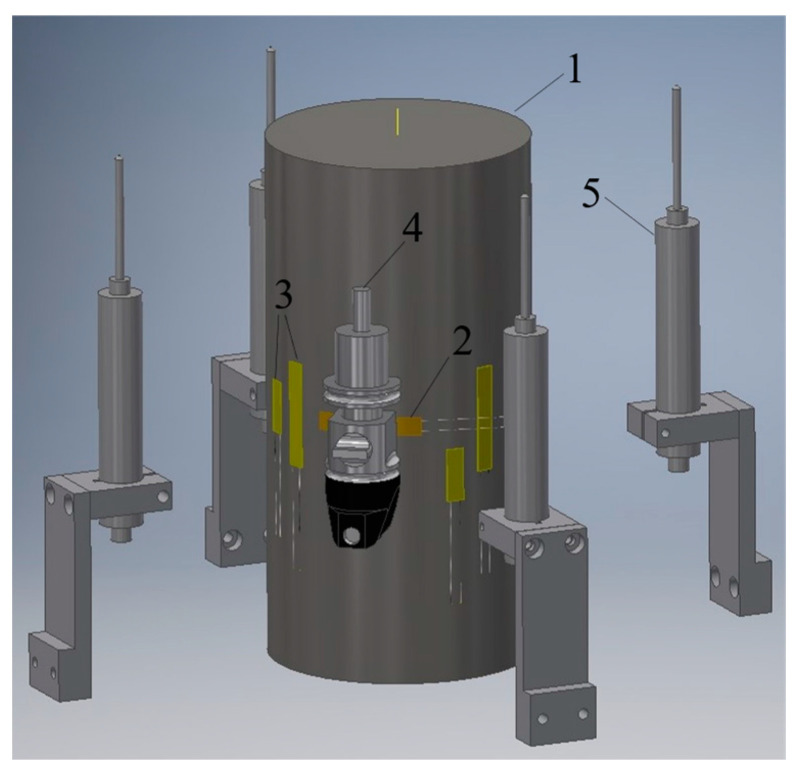
Test setup: 1—cylindrical concrete specimen; 2—transverse SG sensors; 3—longitudinal SG sensors; 4—HAT devices; 5—LDT devices for measuring displacement between testing machine plates, following [22].

The described model of compressed concrete specimen behavior in the transverse direction was experimentally confirmed in [15]. Additionally, experimental investigations of specimen behavior were carried out in transverse and longitudinal directions in [22]. Both elastic and inelastic stages (including concrete post-peak response) were analyzed. Transverse deformations were measured with two strain gauges (SGs) with 60 mm bases located in the specimen central zone. To measure longitudinal deformations, eight 30 mm and 60 mm long SGs and two longitudinal, highly accurate transducers (HATs) with 50 mm measurement bases were used. Additionally, four linear displacement transducers (LDTs) with 50 mm strokes were placed between the testing machine loading plates to obtain an accurate descending branch for the stress–strain diagram. A general view of the test setup is shown in Figure 4.

**Figure 5:**
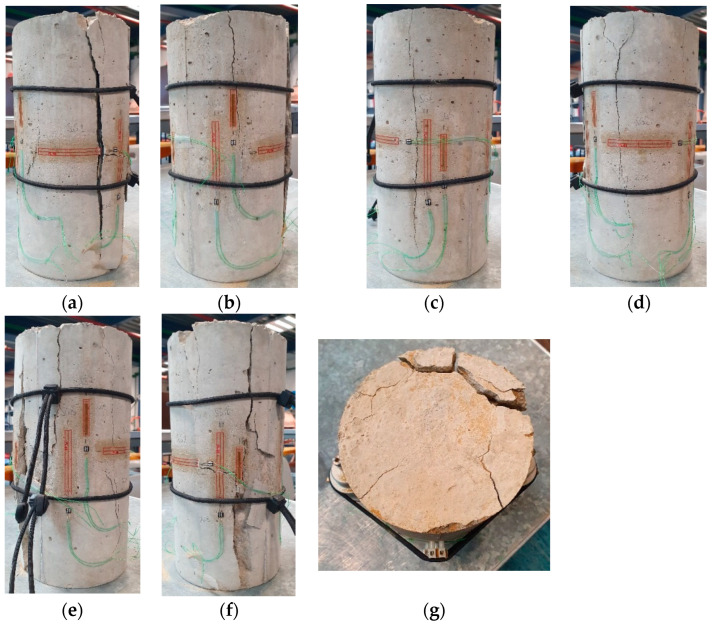
Typical pattern of a cracked specimen: (**a**–**f**)—crack propagation after specimen failure (view from all sides); (**g**)—failure pattern of the specimen upper surface.

According to the obtained experimental data, the development of transverse deformations causes longitudinal cracks in compressed concrete cylindrical specimens. The typical failure pattern with vertical cracks and the internal undamaged cylindrical core, described above, is shown in Figure 5 (following [22]).

The obtained experimental results for transverse and longitudinal deformations are used in further step-by-step analysis.

### 4.2. The Step-by-Step Analysis: Description and Application

**Figure 6:**
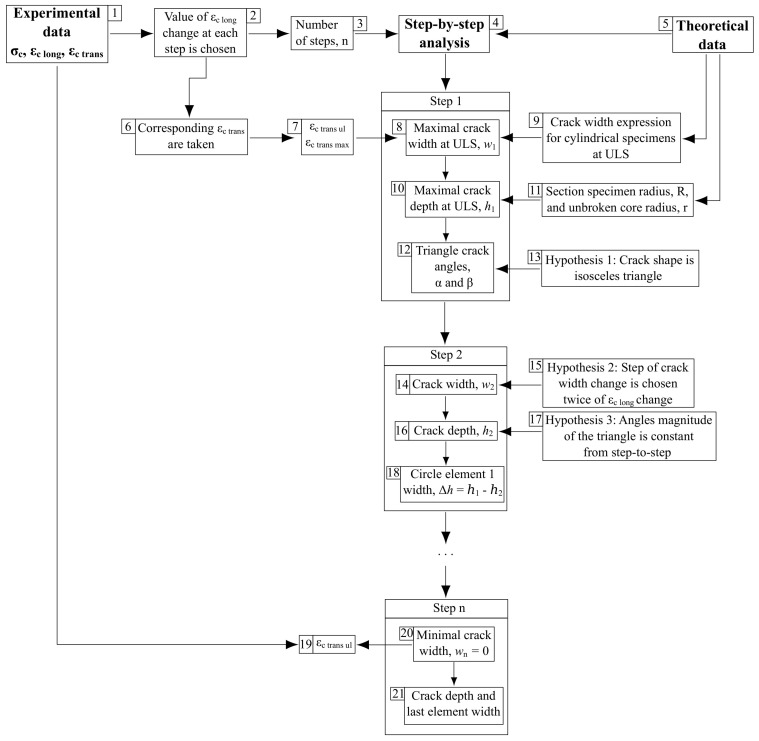
Crack width and depth calculation algorithm in compressed concrete element.

**Figure 7:**
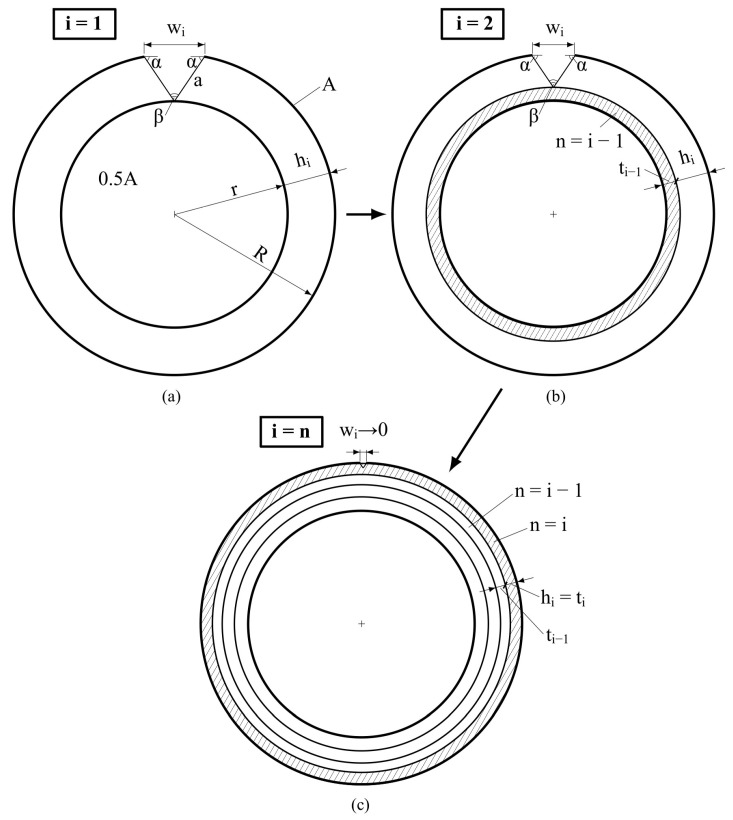
Step-by-step numerical analysis schemes: (**a**) step 1—cracked specimen at the peak stress; (**b**) step 2—the first circle element calculation; (**c**) final step—the last circle element calculation when the crack width tends to zero.

The analysis is based on crack width and depth step calculations, $w_{i}$ and $h_{i}$ (i=1…5 is a step number), defining the undamaged circular element width in the cylindrical specimen section, $t_{i}$, in each calculation step (Figure 6 and Figure 7). Figure 6 presents the calculation algorithm diagram. Schemes of the performed analysis are demonstrated in Figure 7.

As shown in the algorithm diagram (see Figure 6), the calculation runs in reverse order, starting with the specimen limit state ($\sigma_{c}=f_{c}$ and $\varepsilon_{c}=\varepsilon_{c1}$), when the section area of the specimen’s inner undamaged core is equal to half of its initial value [15] (see cell 11 in Figure 6; see also Figure 7a). The calculation ends at the stage corresponding to the crack initiation because of transverse deformations (the end of the specimen’s elastic behavior) when the following parameters are obtained:

– The crack width is theoretically equal to zero;
– The experimental value of the transverse deformation corresponds to concrete tensile strength in the transverse direction, $f_{ct}$ [26] (cells 19–21 in Figure 6).

The following hypotheses are proposed by the authors as a basis for the step-by-step analysis:

1. The crack shape in the horizontal section is an isosceles triangle (see Figure 7).
2. Each calculation step of the crack width, Δw, and depth, Δh, is determined as a function of longitudinal deformation development.
3. The triangle angles, α and β, remain constant from step to step.

At the initial calculation stage, preceding calculation step 1, a certain longitudinal compression strain step is selected equal to $\varepsilon_{c\;long\;i}/1.44$ (1.44 is selected to obtain an integer number of calculation steps) based on the experimental limit value, $\varepsilon_{c1}$ (see cell 2 in Figure 6). This determines the calculation steps number, n, obtained using geometric progression (see cell 3 in Figure 6):

(1) $$n=1+\frac{\log\frac{\varepsilon_{c\;el}}{\varepsilon_{c1}}}{\log\frac{1}{1.44}}$$

For example, for the limit and maximal elastic longitudinal deformation values, $\varepsilon_{c1}=2‰$ and $\varepsilon_{c\;el}=0.5‰$, respectively; the number of steps is *n* = 5. Further, given the longitudinal strain experimental values, $\varepsilon_{c\;long}$, the corresponding transverse strains, $\varepsilon_{c\;trans}$, are taken (see cell 6 in Figure 6). Thus, the basic calculation parameters are determined (see cells 1–3 and 6–7 in Figure 6).

At the next stage, the first step of analysis (i=1) starts (see cells 8, 10, and 12 in Figure 6 and also Figure 7a). At this step, the specimen limit state by transverse deformations is considered. In the beginning, the maximum specimen crack width, $w_{1}=w_{max}$, is calculated using a known expression (Equation (5) in [26], describing concrete tensile behavior in different stress–strain conditions). The crack width calculation is based on the ultimate transverse strains, $\varepsilon_{c\;trans\;ul}$, and the transverse strain corresponding to the maximal crack width at peak stress, $\varepsilon_{c\;trans\;max}$ (see cells 7–9 in Figure 6). Next, the crack depth, $h_{1}=h_{max}$, is calculated: it is equal to the difference between the specimen section radius before loading, R, and after reaching the limit state in the longitudinal direction, r (which is equal to the undamaged core radius) (see cells 10 and 11 in Figure 6 and also Figure 7a). After that, triangle angles α and β are calculated based on hypothesis 1, which determines the crack shape in the horizontal section as an isosceles triangle (see cells 12 and 13 in Figure 6 and also Figure 7a).

The second step of the analysis (i=2) starts with the calculation of the crack width, $w_{2}<w_{1}$ (because of the calculation’s reverse order) using hypothesis 2 (see Figure 7b and cells 14 and 15 in Figure 6). According to the hypothesis, the incremental change in crack width is assumed to be twice the longitudinal strain change (according to the structural phenomenon [15]). In general, the expression for crack width calculations is

(2) $$w_{i}=w_{i-1}/\left(2\cdot 1.44\right)$$

Next, the crack depth, $h_{2}<h_{1}$, is calculated based on hypothesis 3 (see cells 16 and 17 in Figure 6 and also Figure 7b). After that, the width of element 1, $t_{1}$, is calculated—it is equal to the difference between the crack depths from steps 1 and 2 (see cell 18 in Figure 6 and also Figure 7b). In general, the equation for element width calculation has the following form:

(3) $$t_{i-1}=h_{i-1}-h_{i}$$

The process described in step 2 is repeated $\left(n-1\right)$ times. The step-by-step analysis ends with step n (see Figure 7c), at which point, the calculated crack width is theoretically equal to zero, and the transverse strain value is equal to $\varepsilon_{c\;trans\;ul}$ (see cells 19–21 in Figure 6).

### 4.3. Results and Discussion

**Figure 8:**
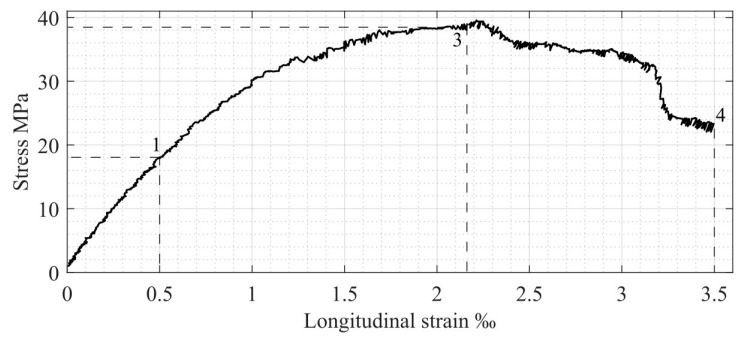
Longitudinal stresses vs. longitudinal strains.

**Figure 9:**
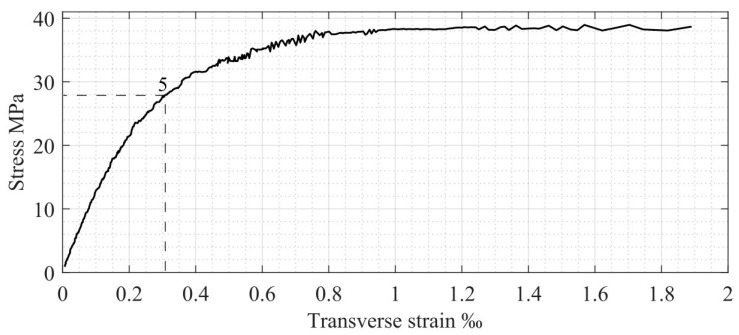
Longitudinal stresses vs. transverse strains.

As described previously, the existing experimental longitudinal and transverse deformations for the compressed concrete cylindrical specimen [22] are used in the present step-by-step analysis. Relationships between stresses and longitudinal strains and stresses and transverse strains (including basic points 1, 3, 4, and 5, described previously in Figure 1) are shown in Figure 8 and Figure 9, respectively.

**Figure 10:**
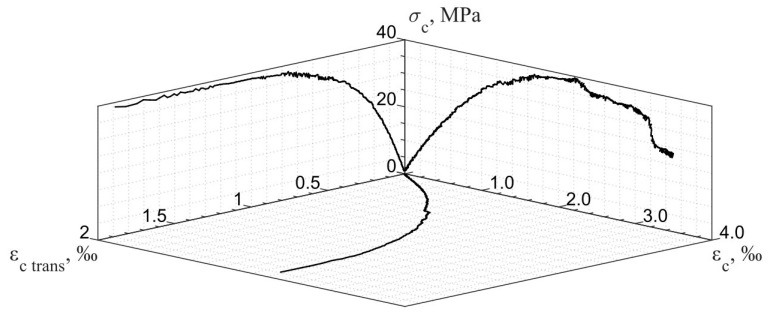
Experimental relationship between longitudinal stresses and longitudinal and transverse strains.

To obtain a more accurate representation of compressed concrete behavior, we propose combining the relationships shown in Figure 8 and Figure 9 (Figure 10). The obtained diagram demonstrates the relationship between the following three dependencies: longitudinal and transverse strain development in a concrete cylindrical specimen ($\sigma_{c}-\varepsilon_{c\;long}$ and $\sigma_{c}-\varepsilon_{c\;trans}$ axis) and the relationship between them ($\varepsilon_{c\;trans}-\varepsilon_{c\;long}$ axis) as a function of compression loading.

Table 2 presents selected experimental data for the initial step-by-step analysis stage. The number of calculation steps is selected to be equal to five, where step 1 corresponds to the concrete limit state. Additionally, the modulus of deformability, $E_{c}$, is calculated for each step based on the experimental data presented in Table 2. The result obtained in step 1 demonstrates a twofold decrease in the modulus magnitude after reaching peak stress (from 36.1 to 18 MPa), which corresponds to the above-mentioned structural phenomenon.

**Figure 11:**
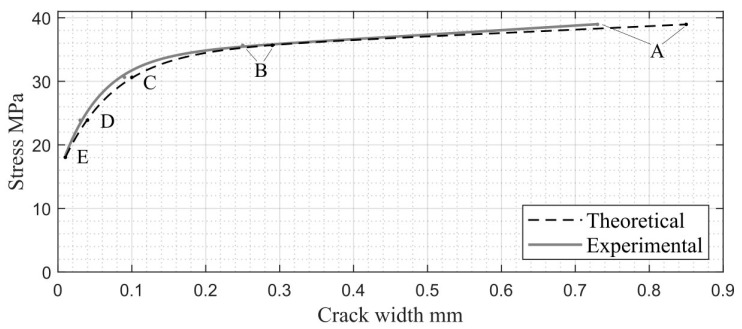
Stresses vs. crack widths due to transverse deformations: A—step 1; B—step 2; C—step 3; D—step 4; E—step 5.

The experimental data presented in Table 2 are used for the step-by-step numerical analysis. According to the above-described analysis algorithm (see Figure 6), the following parameters of compressed concrete behavior are calculated at each step: crack width, $w_{i}$; crack depth, $h_{i}$; and element width, $t_{i}$. The obtained results are presented in Table 3. The table includes a comparison of crack widths, $w^{Theor}$ and $w^{Exper}$, calculated using theoretical deformation values [26] and experimental ones (see Table 2), respectively. A graphical representation of the stress and crack width relationship is shown in Figure 11.

**Figure 12:**
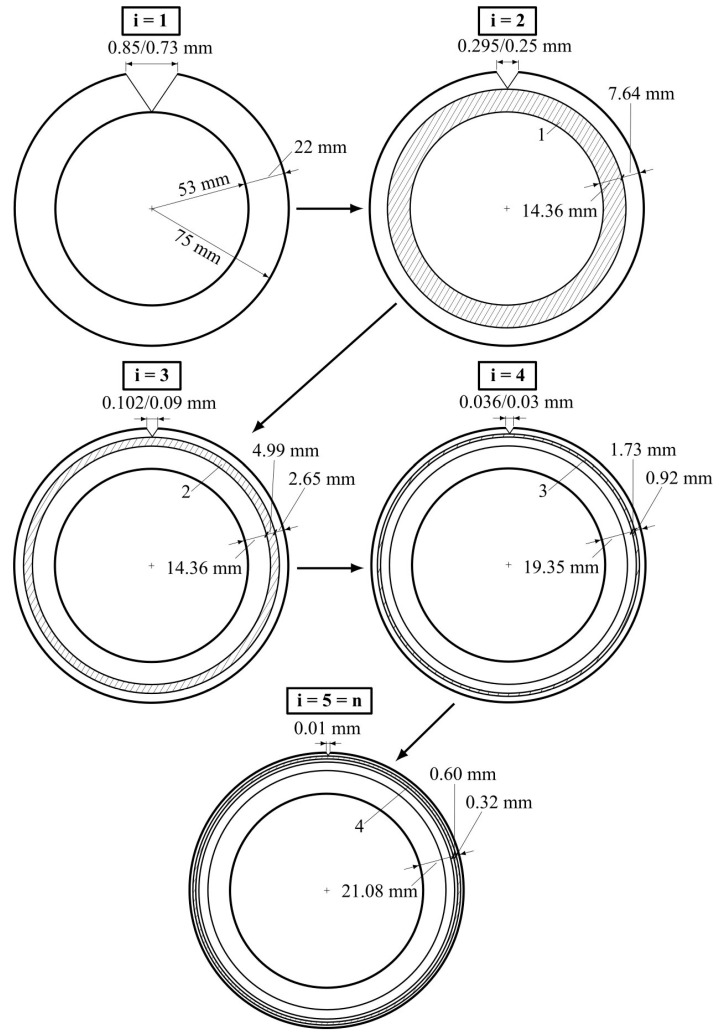
Step-by-step numerical analysis schemes (numerator—theoretical value; denominator—experimental value).

In step 1, the crack width corresponds to the element limit state. In this case, the calculation of $w^{Theor}$ is based on transverse deformation, $\varepsilon_{c\;trans\;max}$: 2−0.2=1.8‰, where 0.2‰ is the theoretical value of the ultimate transverse deformations, $\varepsilon_{c\;trans\;ul}$, and 2‰ is the transverse deformation value corresponding to the maximal crack width. These values correspond to the experimental transverse deformations in steps 5 ($\varepsilon_{c\;trans}=0.156‰$) and 1 ($\varepsilon_{c\;trans}=1.703‰$) (see Table 2), which were used to determine $w^{Exper}$. The obtained relative error, D=14.02 %, shows a good convergence between theoretically and experimentally based results for crack width at the limit state of the compressed concrete element. The same numerical data are shown in Figure 12.

The maximum crack depth, $h_{max}=h_{1}$, is also defined in step 1 as equal to 22 mm (see Table 3 and Figure 12). This value is calculated according to the above-described experimentally verified theoretical model of the concrete cylindrical specimen section at its ULS, based on the structural phenomenon [15] (the specimen section area is two times higher compared with the inner undamaged core one). Correspondingly, the sum of all element widths is

(4) $$\overset{n}{\underset{i=1}{\sum }}t_{i}=h_{1}$$

According to the obtained numerical results, the crack width reaches 0.3 mm in step 2 (this step is the pre-ultimate stage before the element limit state). This value complies with modern code requirements for the maximum crack width in concrete elements [24,25]. At the last calculation step ($\sigma_{ct\;trans}=f_{ct}$), the crack width tends to be zero when transverse deformations correspond to crack initiation (see Table 2 and Figure 9 and Figure 10).

Thus, a complex numerical analysis of compressed concrete element behavior was conducted based on an investigation of crack development caused by transverse deformations. The research outcomes justify the efficiency of the proposed approach for the investigation of compressed concrete in the limit state by using its behavior parameters not only in the longitudinal direction but also in the transverse one.

### 4.4. Example of Using the Step-by-Step Analysis Algorithm

The purpose of this example is to identify the limit state of a cylindrical compressed concrete specimen of 300 mm in height and 150 mm in diameter by transverse deformations. The following theoretical data, as well as the experimentally obtained data, are provided for step-by-step analysis:$\;\varepsilon_{c\;trans\;ul}^{Theor}=0.200‰$, $\varepsilon_{c\;trans\;ul}^{Exper}=0.156‰$, $\varepsilon_{c\;trans\;max}^{Theor}=2.000‰$, $\varepsilon_{c\;trans\;max}^{Exper}=1.700‰$, R=75 mm, r=53 mm.

In the first analysis step (i=1), the maximum specimen crack width is calculated:

(5) $$w_{1}^{Theor}=\frac{\left(2.000-0.200\right)\cdot \pi \cdot 150}{1000}=0.85\;\mathrm{mm}$$

(6) $$w_{1}^{Exper}=\frac{\left(1.700-0.156\right)\cdot \pi \cdot 150}{1000}=0.73\;\mathrm{mm}$$

The maximum crack depth is $h_{1}=75-53=22\;\mathrm{mm}$. The triangle angles of crack shapes α and β are calculated, where the hypotenuse lengths are $a^{Theor}=22.0041\;\mathrm{mm}$ and $a^{Exper}=22.0030\;\mathrm{mm}$:

(7) $$\alpha =\cos^{-1}\left(\frac{w}{2a}\right);\;\beta =180{}^{\circ}-2\alpha$$

The obtained results are shown in Table 4.

In the second analysis step (i=2), the crack width is calculated according to Equation (2):

(8) $$w_{2}^{Theor}=\frac{0.85}{2\cdot 1.44}=0.30\;\mathrm{mm}$$

(9) $$w_{2}^{Exper}=\frac{0.73}{2\cdot 1.44}=0.25\;\mathrm{mm}$$

The crack depth at this step is

(10) $$h_{2}=\frac{w_{2}}{2\cos\alpha }\cdot \cos\left(\frac{\beta }{2}\right)=7.64\;\mathrm{mm}$$

The first element width is calculated using Equation (3):

(11) $$t_{1}=22-7.64=14.36\;\mathrm{mm}$$

Steps 3, 4, and 5 of the analysis repeat procedures described in step 2. The results of the analysis are presented in Table 3.

Thus, in the calculation example, the final theoretical and experimental values of crack width and depth in the compressed concrete element are obtained at all stages of its performance, including the limit state. The next stage compares the specified values with modern code requirements for the final formulation of the limit state calculation based on transverse deformations in a real element.

It should be noted that a compressed element limit state by the crack depth, caused by transverse deformations, is proposed for the first time and should be associated with a protective coating of reinforcement. The maximum crack depth obtained in the example is equal to 7.64 mm, which corresponds to the maximum crack width value required by modern codes [24,25]. This depth value is usually acceptable for compressed elements where the concrete coating layer is at least 1 cm. This parameter is especially critical for thin-walled RC structures, particularly for shells.

## 5. Conclusions

A complex analysis of compressed concrete limit states was conducted based on the existing theoretical and experimental data, including longitudinal and transverse deformations and longitudinal crack width and depth. Based on the obtained results, the following conclusions are evident:

1. A numerical investigation of compressed concrete element behavior in the longitudinal direction based on theoretical and experimental results was conducted, and the high accuracy of the theoretical stress vs. longitudinal strain relationship was justified. This, in turn, formed the basis of a numerical investigation of compressed concrete limit state in the transverse direction.
2. A new approach for the simultaneous consideration of compressed concrete elements limit states based on transverse deformations was proposed. A corresponding graph of concrete behavior in the longitudinal and transverse directions was used for this analysis.
3. For the first time, reverse-order step-by-step analysis was proposed, starting with the compressed concrete element’s ULS and finishing at the end of its elastic stage, which, at the initial stage, allowed us to obtain data for the width and depth of cracks caused by transverse deformations.
4. A diagram simultaneously representing specimen behavior in the longitudinal and transverse directions was proposed as a tool for the comprehensive study of compressed concrete performance.
5. For the first time, the limit state by the crack depth was introduced as a function of crack width development. This allows for the theoretical determination of the minimum concrete coating value for reinforcement in compressed elements (or in the compressed concrete zone of bending elements), which is currently proposed in modern codes as an empirical value.

This research reveals additional potential for further limit state investigations based on the depth and width of cracks caused by transverse deformations in compressed concrete elements.

## Figures and Tables

**Table 1:** Concrete elastic and plastic potentials and relationships between them.

Specimen	Elastic Potential, kPa		D, %	Plastic Potential, kPa		D, %	$\frac{U_{el}^{Num}}{U_{pl}^{Num}}$	Average $\frac{U_{el}^{Num}}{U_{pl}^{Num}}$
	Numerical	Experimental		Numerical	Experimental			
Sp3.1	30.86	32.87	6.13	54.58	65.81	17.06	0.52	0.51
Sp3.2	37.27	38.72	3.74	75.62	63.13	16.52	0.49	
Sp3.3	36.09	36.69	1.64	72.07	71.95	0.17	0.50	
Sp4.1	18.38	18.87	2.60	35.62	39.54	9.91	0.52	
Sp4.2	16.50	17.14	3.73	31.90	37.54	15.02	0.52	
Sp4.3	20.68	21.72	4.79	40.41	37.07	8.27	0.51	

Note: $U_{el}^{Num}$ and $U_{pl}^{Num}$ are concrete elastic and plastic potentials, calculated based on numerical results; $U_{el}^{Exper}$ and $U_{pl}^{Exper}$ are concrete elastic and plastic potentials, calculated based on experimental results; D is relative error.

**Table 2:** Basic experimental data.

Step Number	$\sigma_{c}$, MPa	$\varepsilon_{c\;long}$, ‰	$E_{c}$, MPa	$\varepsilon_{c\;trans}$, ‰
1	38.96	2.16	18.0	1.703
2	23.88	0.72	33.1	0.230
3	30.63	1.04	29.5	0.373
4	23.88	0.72	33.1	0.230
5	18.05	0.50	36.1	0.156

**Table 3:** Numerical results of the step-by-step analysis.

Step Number	$w^{Theor}$, mm	$w^{Exper}$, mm	D, %	h, mm	t, mm
1	0.85	0.73	14.02	22.00	14.36
2	0.30	0.25		7.64	4.99
3	0.10	0.09		2.65	1.73
4	0.04	0.03		0.92	0.60
5	0.01	0.01		0.32	0.32

**Table 4:** Theoretical and experimental values of triangle angles *α* and *β*.

	Theoretical °	Experimental °
*α*	88.90	89.05
*β*	2.21	1.90

## Data Availability

Data are contained within the article.
